# Laboratory-acquired infections in Canada from 2016 to 2021

**DOI:** 10.14745/ccdr.v48i78a02

**Published:** 2022-07-07

**Authors:** Maryem El Jaouhari, Megan Striha, Rojiemiahd Edjoc, Samuel Bonti-Ankomah

**Affiliations:** 1 Immunization Branch, Public Health Agency of Canada, Ottawa, ON; 2 Health Security and Regional Operations Branch, Public Health Agency of Canada, Ottawa, ON; 3 Health Promotion and Chronic Disease Prevention Branch, Public Health Agency of Canada, Ottawa, ON

**Keywords:** laboratory-acquired infection, LAI, laboratory exposures, human pathogens and toxins

## Abstract

incidents that result in an exposure to human pathogens and toxins can lead to laboratory-acquired infections or intoxications (LAIs). These infections can pose a risk to the public as well, should person-to-person transmission occur outside the laboratory after an LAI. Understanding factors that contribute to exposure incidents involving LAIs may contribute to ways to mitigate future occurrences to ensure the safety of laboratory workers and the communities in which they work. This paper describes nine exposure incidents resulting in LAIs that occurred in Canada from 2016 to 2021. Of the nine cases, most affected people had both high level of education and years of experience working with pathogens. There were varying laboratory types and activities where *Salmonella* spp. and *Escherichia coli* accounted for six out of the nine cases. Procedural issues, personal protective equipment issues and sharp-related incidents were the most cited root causes. From this information, it is clear that regular training (even of experienced staff), clear and accurate standard operating procedures, proper hygiene (especially with *Salmonella* spp. and *E. coli*) and recognition of exposure incidents at the time of occurrence are important in preventing future LAIs. Only regulated laboratories working with risk group 2 or higher organisms are required to report exposures and LAIs to the Laboratory Incident Notification Canada surveillance system. Because of the small sample size, results and inferences are based on descriptive analyses only.

## Introduction

Working with human pathogens and toxins (HPTs) in a laboratory setting is an inherently risky activity, particularly when working with higher-risk group pathogens and toxins. While safety protocols, practices and equipment are all utilized to keep laboratory workers safe, accidents, failures or other incidents can still occur. Incidents that result in an exposure to HPTs can lead to laboratory-acquired infections or intoxications (LAIs). These infections can pose a risk to the public as well, should person-to-person transmission occur outside the laboratory after a LAI.

The Public Health Agency of Canada’s Centre for Biosecurity contributes to the Agency’s efforts to protect the health, safety and security of the Canadian public against the risks posed by human pathogens and toxins. The Centre for Biosecurity launched the Laboratory Incident Notification Canada (LINC) surveillance system in late 2015. Beginning in 2016, licensed facilities are required to submit reports to LINC detailing any laboratory incidents involving HPTs of risk group (RG) 2 or higher, in accordance with the *Human Pathogens and Toxins Act*. Reports submitted to LINC may describe exposure or non-exposure incidents, where exposures are defined as an incident that could have resulted in intoxication/infection or has resulted in a suspected or confirmed LAI ((1,2)). A more general overview of LINC, including detailed descriptions of the incidents reported to LINC, is available in the annual reports (2016 to present) ((3–7)).

A search of the literature found nine LAI case reports that highlight key risk factors (none were from Canada). Results from one study indicated that the lack of adherence to standard biosafety procedures was a major factor in LAIs ((8)). Several studies found that improper use of personal protective equipment (PPE) was associated with the occurrence of LAIs ((8–12)). Additionally, the lack of respiratory PPE was the most common risk factor among 16 cases ((11)). Other risk factors identified in the literature include improper use of laboratory equipment ((13)), working with needles ((14–16)), lack of hygienic practices ((7,12)) and insufficiently trained staff ((13,14)). Among the studies reviewed, the most common pathogens involved in LAIs were *Salmonella* spp., *Brucella* spp., *Staphylococcus aureus*, *Escherichia coli*, *Neisseria meningitidis* and *Vaccinia virus*. Additionally, recent analysis of LINC (*forthcoming*) exposure reports found that standard operating procedure (SOP)-related issues were a significant risk factor to the overall increase in exposure events in Canadian laboratories ((15)).

This study describes nine cases of LAIs that occurred in Canada between 2016 and 2021. Data were extracted from the LINC surveillance system for all confirmed LAI reports. The objective of this study is to describe the LAIs and to identify potential risk factors associated with LAIs in Canada.

## Results

Between 2016 and 2021, nine LAIs were reported to LINC. During the same period, 322 exposure incidents that could have resulted, or did result, in LAIs were reported to LINC. Multiple individuals can be exposed during a single incident, and in total, 668 people were exposed in the 322 incidents. Therefore, 1.3% of people exposed ended up developing an LAI (and less than 3% of incidents).

All nine LAIs occurred in technicians, students and laboratory aids (**Table 1**). Most of the LAIs occurred in people who had either a high level of education or many years of laboratory experience; and sometimes both. The median number of years of experience was six years for the eight people for whom the information is known. None of these LAIs led to secondary infections.

**Table 1:** Descriptions of each of the nine confirmed laboratory-acquired infections in the Laboratory Incident Notification Canada surveillance system, Canada, 2016–2021

Variable	Case 1	Case 2	Case 3	Case 4	Case 5	Case 6	Case 7	Case 8	Case 9
Role	Student	Technician	Technician	Technician	Technician	Technician	Technician	Aide	Student
Highest degree	Master’s Degree	Technical diploma	Bachelor’s degree	Bachelor’s degree	Bachelor’s degree	Technical diploma	Bachelor’s degree	High School diploma	Bachelor’s degree
Years of experience	Fewer than 5	Fewer than 5	Unknown	Fewer than 5	10–20	5–10	5–10	20 or more	Fewer than 5
Laboratory type	Academic	Hospital	Government Public Health	Hospital	Government Public Health	Hospital	Academic	Hospital	Government (other)
Main work activity	*In vivo* animal work	Microbiology	Microscopy	Microbiology	Microbiology	Microbiology	Animal care	Maintenance	Microbiology
Biological agent	*Staphylococcus aureus*	*Salmonella* spp.	*Salmonella* spp.	*Brucella* spp.	*Salmonella* spp.	*E. coli*	*Vaccinia virus*	*Salmonella* spp.	*E. coli*
Risk group	RG2	RG2	RG2	RG2 or RG3	RG2	RG2	RG2	RG2	RG2
Exposure route	Inoculation	Ingestion (presumed)	Ingestion	Inhalation	Ingestion	Ingestion	Inoculation	Ingestion	Absorption
Exposure cause	Sharps	Unknown	Equipment, PPE, procedural	PPE, procedural	Unknown	Procedural	Sharps, procedural	PPE, Procedural	Spill, equipment, procedural
Exposure recognized at time?	Yes	No	No	Yes	No	No	Yes	No	Yes
Immediate first aid?	Yes	N/A	N/A	No	N/A	N/A	Yes	N/A	No
Acute illness	Yes	Yes	Yes	No (seroconversion)	Yes	Yes	Yes	Yes	Yes
Medical consult (fewer than 8 days)	No	Yes	No	Yes	Yes	Yes	Yes	No	Yes
Medical consult (8 or more days)	Yes	Yes	Yes	Yes	No	Yes	Yes	Yes	Yes
Occupational health consult (fewer than 8 days)	No	Yes	No	Yes	No	Yes	Yes	No	No
Occupational health consult (8 or more days)	Yes	Yes	No	No	No	Yes	No	Yes	No
Prophylaxis	Yes	N/A	N/A	Yes	N/A	N/A	Yes	N/A	No
Drug treatment	Yes	No	Yes	No	No	No	No	Yes	No
Recovery time	Unknown	8–14 days	14 or more days	N/A	8–14 days	Fewer than 8 days	Unknown	14 or more days	8–14 days

Abbreviations: N/A, not applicable; PPE, personal protective equipment; RG, risk group

Additionally, there were a range of laboratory types involved (Table 1), indicating that LAIs can occur in different settings. The most common laboratory activities associated with these LAIs were microbiology (n=5), followed by animal work (n=2), microscopy (n=1) and maintenance (n=1).

Consistent with previously published articles, the agents associated with the nine LAIs were *Salmonella* spp. (n=4), *E. coli* (n=2), *S. aureus* (n=1), *Brucella* spp. (n=1) and *Vaccinia virus* (n=1).

Of the two animal-related incidents, both LAIs resulted from inoculation via sharps-related exposure. The other seven incidents were a mix of ingestion (n=5), absorption (n=1) and inhalation (n=1). In addition to the two sharps-related incidents, the other commonly cited root causes were procedural, PPE, equipment or spill-related.

Of the nine confirmed LAIs, only four exposure incidents were recognized as such at the time of the event. The other five exposure incidents were retrospectively identified, after the workers became ill.

Of the four LAIs where the exposure incident was recognized at the time, two people received immediate first aid attention and three of the four received prophylaxis. In addition, three of four people consulted a medical professional within seven days of the exposure. Unfortunately, even with these preventative interventions, three of the four people became acutely ill, while the fourth tested positive for seroconversion (indicating an asymptomatic infection).

Of the five LAIs that stemmed from unrecognized exposure events, all five became acutely ill and sought medical and/or occupational health consultations, after which an LAI was identified and reported to LINC. These illnesses led to investigations into whether the illnesses were related to exposure to HPTs. Exposure incidents that led to the LAIs were then retroactively identified where possible, working backwards from the date of illness using the incubation period of the HPT.

Three of nine people received drug treatment for their illness. While the recovery period varied, it often took more than a week (n=5).

## Discussion

The primary objectives for this study were to describe the nine LAIs that have occurred in Canada between 2016 and 2021 and to identify potential risk factors associated with these incidents. Because of the small sample size, results and inferences are based on descriptive analyses only. In addition, only regulated laboratories working with RG2 or higher organisms are required to report exposures and LAIs to LINC. Exposures and LAIs stemming from work with primary specimens (such as blood or other samples from patients) are not required to be reported to LINC, although it is strongly recommended. All nine LAIs described here are from mandatory reporting situations.

Most of the people with LAIs in this study had either a high level of education, many years of laboratory experience, or both. This suggests that inexperience or lower levels of education may not be a risk factor for LAIs. Regular training and reviewing of standard operating procedures with staff, both new and experienced, is key to preventing exposure incidents and LAIs.

Additionally, the range of laboratory types (academic, hospital and government) and activity types (microbiology, animal care, etc.) reported suggest that work in any laboratory type and any laboratory activity could lead to a LAI.

As seen in the literature, *Salmonella* spp. and *E. coli* were the most common HPTs involved in LAIs. Further investigation into the reasons and mechanisms behind the association of these two pathogens and LAIs is recommended.

Many underlying causes are mentioned amongst the nine reports, but procedural issues are cited in most of them. Having detailed, accurate and up-to-date SOPs in place is critical, as is the ongoing training and refreshing of staff on the proper SOPs for their activities. In addition, the use of appropriate PPE is always critical to protect laboratory personnel from infections. Procedural issues may include a lack of an appropriate SOP, following a SOP inappropriate for the activity or failing to follow the SOP as written. The PPE-related incidents may include lack of PPE, misuse of PPE or a failure or malfunction of the PPE. Similarly, equipment issues can include misuse of equipment or a failure or malfunction of the equipment.

It is important to recognize and respond to exposure events when they occur in order to prevent LAIs and community transmission. Of the nice LAIs identified, fewer than half of the exposure incidents were recognized as such at the time of the event. This is problematic, as failure to identify exposures at the time of the incident does not enable implementation of recommended procedures. Laboratories have specific procedures in place to respond to accidental exposures, including first aid, immediate medical consultation, prophylaxis and measures to prevent spread should a LAI occur (such as quarantine). When an exposure is overlooked, none of these preventative actions can take place, increasing the likelihood that an LAI will occur. Furthermore, these events are then more likely to lead to community transmission as a person may be contagious without knowing until they develop signs and symptoms of an LAI.

## Conclusion

There have been nine reported LAIs in Canada in the last five and a half years, none of which led to community spread. *Salmonella* spp. and *E. coli* are two HPTs of concern when it comes to LAIs. It is important for laboratories to train all staff on the proper procedures for their duties, with regular retraining, including updates as soon as possible when procedures change. In addition, exposure incidents should always be reported immediately, with guidelines for actions after exposure followed thoroughly to prevent LAIs and community spread.
